# Driving pathways of university teachers’ professional development: the roles of perceived team leadership, innovative self-efficacy, and organizational innovation climate

**DOI:** 10.3389/fpsyg.2026.1769140

**Published:** 2026-04-07

**Authors:** Xiaona Liu, Chang Liu, Yu Sun, Hu Gao, Chuang Xu

**Affiliations:** 1 Teacher Education Department, Hengshui University, Hengshui, China; 2 Foreign Languages Department, Hengshui University, Hengshui, China; 3 Marxism Department, Hengshui University, Hengshui, China; 4 Shaanxi Vocational Academy of Art, Xi'an, China; 5 School of Marxism, Hunan Institute of Technology, Hengyang, Hunan, China

**Keywords:** innovative self-efficacy, organizational innovation climate, perceived team leadership, Social Exchange Theory, teacher professional development

## Abstract

**Introduction:**

The professional development of individuals within organizations is a crucial component in shaping organizational competitive advantage. Although existing research indicates that leadership styles can significantly influence individual professional development, there is limited understanding of the mechanisms and boundary conditions governing the relationship between perceived team leadership and individual professional development.

**Methods:**

Based on valid survey questionnaires from 655 university teachers in Hebei Province, China, this article constructs and tests a moderated mediation model.

**Results:**

Empirical results demonstrate that: perceived team leadership has a significant positive impact on university teachers’ professional development; innovative self-efficacy plays a partial mediating role in the relationship between perceived team leadership and university teachers’ professional development; organizational innovation climate positively moderates all paths within the mediation framework.

**Conclusion:**

This finding reveals the interactive effects of “context-individual-behavior,” extends the application boundaries of Social Exchange Theory in educational organizations, and demonstrates that leadership effectiveness also depends on organizational climate support. This article elucidates the mechanism by which perceived team leadership influences the professional development of university teachers, providing practical insights for university administrators, such as enhancing team leadership, fostering organizational innovation climate, and activating teachers’ intrinsic motivation for development.

## 1 Introduction

In the era of the knowledge economy, the professional growth and innovative capabilities of organizational members have increasingly become the core driving force for the sustainable development of various types of organizations. As key agents of knowledge creation, dissemination, and application within organizations, the professional development of individuals not only concerns their own career achievements but also influences the innovation and performance outcomes of the entire organization. However, individuals currently face numerous challenges in their professional growth, such as occupational burnout, insufficient motivation for innovation, and imperfect organizational support systems. These issues severely constrain the full utilization of individual potential and the achievement of organizational goals. Particularly during critical periods of organizational transitional development, how to effectively stimulate individuals’ intrinsic motivation and innovative vitality has become a significant proposition for modern organizational governance.

Research in the field of organizational psychology indicates that individual professional development is influenced by multi-level factors. At the individual level, psychological resources such as professional identity (Yang and Zhou, 2025), career orientation, self-efficacy (Xiong and Zhu, 2013), professional literacy, and reflective ability (Du and Chang, 2018); at the environmental level, external contexts such as social demand, organizational culture, and colleague interactions are also important antecedents (Du and Chang, 2018; Avalos, 2011; Prenger et al., 2017). In recent years, scholars have begun to focus on the role of leadership in promoting individual development. For example, Kilag and Sasan (2023) showed that instructional leadership plays a vital role in individual professional development and growth; Karacabey et al. (2022) confirmed that leaders can influence individual professional learning and growth through the mediating effects of collective efficacy and trust, this suggests that deficiencies or the absence of leadership may severely hinder individual development (Mubarak et al., 2025). However, existing research has mostly focused on the direct influence of formal leadership roles, while paying less attention to the perception mechanisms of informal leadership at the team level.

Perceived team leadership, as a shared cognition, can enhance the willingness for collaboration and the frequency of resource exchange among members. According to the reciprocity principle of Social Exchange Theory, team leadership actively provides individuals with more tangible or intangible resources. After individuals perceive these supportive and caring behaviors, they feel obligated to repay the leader, thereby adopting more active and proactive behaviors in their work (Yao et al., 2020; Xuecheng et al., 2022). Individual professional development is a proactive behavior adopted by individuals; essentially, it is a product of the social exchange relationship between perceived team leadership and individuals (Ma, 2011). When individuals perceive support from team leadership, it stimulates their intrinsic motivation to follow reciprocity norms, thereby repaying with positive behaviors (Layek and Koodamara, 2024; Cao et al., 2021).

However, the transformation from willingness to behavior sometimes depends on the mediating role of individual psychological resources. In this process, innovative self-efficacy acts as a key psychological mechanism, playing a bridging function. When individuals feel trusted and empowered during benign interactions with leaders, their internal beliefs gradually establish (Li et al., 2025). This psychological empowerment stimulated by organizational support not only enhances individuals’ confidence in facing challenges but also increases the likelihood of individuals converting their willingness to repay into actual action. In other words, within an interaction relationship based on trust and reciprocity, individuals’ innovative self-efficacy is further reinforced, prompting them to demonstrate greater initiative and creativity in their work.

It is worth noting that the quality and outcome of social exchange relationships are often subject to the moderation of situational factors. Even if individuals possess high innovative self-efficacy, whether this belief can be put into practice is still profoundly influenced by the organizational innovation climate (Xu et al., 2022). Organizational innovation climate, as a shared perception, reflects the common understanding of organizational members regarding an innovation-supportive environment, embodying the organization’s realistic performance in resource investment, management support, tolerance mechanisms, and innovation incentives. In an environment with a high organizational innovation climate, individuals are more inclined to interpret leadership support behaviors as signals of the organization’s overall support for innovation, thereby reinforcing their willingness to repay and their behavioral investment. Conversely, in a low organizational innovation climate, even if individual leaders demonstrate supportive postures, individuals may inhibit innovation attempts due to concerns about conservative organizational culture or performance risks, leading to a disruption in the social exchange chain (Kundu and Roy, 2023). Therefore, the organizational innovation climate not only influences individuals’ interpretation of leadership behaviors but also determines whether they dare to translate enhanced innovative self-efficacy into actual professional development actions.

In summary, although research on the relationship between leadership and teacher professional development has received attention, existing research has not yet systematically revealed: how perceived leadership at the team level influences individual professional development through the psychological mechanism of innovative self-efficacy, and how organizational innovation climate moderates this mediation process. Based on Social Exchange Theory, this study focuses on the complex interactive effects among perceived team leadership, innovative self-efficacy, organizational innovation climate, and individual professional development behaviors, aiming to construct a moderated mediation model. This study uses teachers from local universities in China as a sample to test the above model, providing theoretical bases and practical guidance for enhancing individuals’ professional capabilities.

## 2 Theoretical foundation

This study uses Social Exchange Theory as the core framework to systematically explain the logic linking the research variables. The central proposition of this theory is that interpersonal and organizational interactions are essentially processes of resource exchange grounded in norms of reciprocity. When one party, such as team leaders, provides another party, such as university teachers, with valuable resources including goal guidance, trust-based empowerment, emotional support, and resource coordination, the receiving party develops a psychological sense of obligation to reciprocate and, in order to maintain balance in the exchange relationship, responds with positive attitudes and behaviors (Blau, 1964). Accordingly, this study conceptualizes perceived team leadership as teachers’ subjective cognition of the social resources invested by leaders, innovative self-efficacy as the internal psychological resource accumulated by teachers during the exchange process, university teachers’ professional development as the substantive reciprocating behavior through which teachers fulfill their reciprocal obligation, and organizational innovation climate as a shared situational boundary condition affecting the efficiency of resource conversion. In this framework, the mediating role of innovative self-efficacy follows the path logic of “external resource input-psychological capital accumulation-reciprocal behavioral output.” In the first half of the path, team leaders’ support functions as a form of “social investment” that conveys signals of competence recognition and innovation support to teachers, thereby facilitating the internalization of external support into positive evaluations of their own innovative capability and enhancing innovative self-efficacy (Zhang et al., 2024). In the second half of the path, innovative self-efficacy serves as a psychological driving force that supports teachers in overcoming challenges in professional development, such as teaching reform and research exploration, and in proactively transforming their willingness to reciprocate into actual action, thus completing the social exchange cycle (Afan et al., 2026). At the same time, perceived team leadership can also stimulate teachers’ affective reciprocity motivation through a direct path, directly encouraging them to participate proactively in professional development activities without the mediation of psychological capital (Modliba et al., 2024). Meanwhile, as a shared perception reflecting an organization’s error tolerance mechanisms, innovation incentives, resource allocation, and culture of open collaboration, organizational innovation climate simultaneously exerts moderated mediation effects on all three paths above. In the first half of the mediation path, it acts as an “amplifier of psychological capital conversion”: under a strong innovation climate, teachers interpret leadership support as a consistent organizational signal supporting innovation, which amplifies the resource value of leadership support and strengthens the efficiency with which external resources are converted into innovative self-efficacy; under a weak innovation climate, teachers’ concerns about conservative organizational institutions and innovation risks lower their expectations of reciprocity and weaken this first-half path (Safdar et al., 2024). In the second half of the mediation path, it acts as a “catalyst for behavioral conversion”: under a strong innovation climate, well-developed tolerance and incentive mechanisms reduce teachers’ perceived innovation risks and strengthen their expectations of reciprocity, thereby promoting the efficient conversion of innovative self-efficacy into professional development behavior; under a weak innovation climate, environmental constraints suppress teachers’ willingness to act, causing a rupture in the exchange chain at the stage where psychological capital is converted into behavior (Modliba et al., 2024). In the direct path, it serves as a “safeguard mechanism for direct exchange”: under a strong innovation climate, leadership support is viewed as part of the organization’s overall innovation orientation, amplifying the perceived value of resources and strengthening teachers’ motivation to reciprocate; under a weak innovation climate, leadership support is interpreted as individualized preferential treatment and is therefore insufficient to offset the inhibitory effect of environmental constraints on reciprocity motivation. In this way, organizational innovation climate dynamically influences the strength of the entire mediation effect and forms an interaction model of “situation-individual-behavior.”

## 3 Research hypotheses

### 3.1 The impact of perceived team leadership on university teachers’ professional development

Perceived Team Leadership refers to the overall subjective evaluation by team members of the abilities and behaviors demonstrated by their team leaders in guiding, supporting, and motivating team members. Unlike objective leadership behavior, perception emphasizes how individuals understand and experience the supportive behaviors of leaders; this subjective judgment plays a critical role in organizational interactions. In university organizations, perceived team leadership acts as a vital perceptual organizational support resource, providing teachers with essential resource support conducive to their professional development and growth.

This study aims to explore the impact of perceived team leadership on university teachers’ professional development. Teacher professional development refers to the process in which university teachers continuously improve their knowledge, skills, and attitudes throughout their careers to enhance teaching quality and adapt to changes in the educational environment (Wu and Ke, 2026). According to the reciprocity principle of Social Exchange Theory, when university teachers perceive support and care from team leaders, they in fact obtain a form of socio-emotional resource that goes beyond the formal contract, and this resource triggers a sense of obligation to reciprocate, leading them to respond with positive behavior (Madison et al., 2025). Teachers’ professional development behaviors, such as teaching innovation, research output, and social service, are precisely the primary forms through which teachers, as knowledge workers, reciprocate (Karakus et al., 2024). The positive effect of such leadership perception on employee behavior has also been verified in other organizational contexts. For example, inclusive leadership promotes project success by enhancing employees’ psychological empowerment and psychological safety (Khan et al., 2020), and the underlying mechanism is likewise an exchange of resource input and reciprocal behavior。Meanwhile, a high level of perceived team leadership means that teachers believe leaders can coordinate resources from multiple parties and thereby create more opportunities for their learning and development (Liang et al., 2026). Conversely, when perceived team leadership is low, such as when direction is lacking, communication is poor, or support is insufficient, teachers may experience isolation and frustration, which suppresses the possibility of their professional development. Existing research has found that the stronger perceived team leadership is, the higher the level of university teachers’ professional development is (Afrianty et al., 2025), further confirming the important role of perceived leadership support in driving teachers’ growth. Therefore, from the perspective of Social Exchange Theory, perceived team leadership can be regarded as a valuable socio-emotional support resource that becomes an important psychological driver of university teachers’ professional development by activating teachers’ motivation to reciprocate. By contrast, theories that focus only on leadership behavior itself may overlook the internal transformation of emotion and motivation triggered by perception. What truly promotes teachers’ professional development is not only leaders’ actual behavior, but also how such behavior is interpreted by teachers. Based on the above analysis, this study proposes the following hypothesis:

*H1*: Perceived team leadership positively affects university teachers' professional development.

### 3.2 The mediating role of innovative self-efficacy

Innovative Self-Efficacy refers to an individual’s belief in their ability to produce creative outcomes (Ding and Xie, 2026). According to Social Exchange Theory, the supportive behavior of team leadership perceived by university teachers can be seen as a form of social investment, through which leaders invest trust, empowerment, and emotional resources in teachers. Such investment activates teachers’ willingness to reciprocate, and the premise of reciprocity is that teachers believe they are capable of completing valuable innovative behaviors. Therefore, innovative self-efficacy becomes a crucial psychological mechanism linking external investment to subsequent reciprocal behavior (Al-Mahdy and Elwakil, 2026).

Perceived team leadership positively affects university teachers’ innovative self-efficacy. From the perspective of social exchange, when teachers perceive leaders’ inclusiveness, respect, and support, what they receive is not only emotional resources, but also positive feedback regarding their self-worth. This signal of recognition is internalized into teachers’ confidence in their own abilities, thereby enhancing innovative self-efficacy. This can be explained from the following perspectives. China is a country with a relatively strong collectivist culture, where leaders hold high symbolic significance within an organization; their supportive postures are more easily viewed by teachers as signals of competency recognition (Tao, 2025). Similarly, research on proactive personality indicates that individual psychological traits influence innovative work behavior via the mediating role of work engagement (Mubarak et al., 2021), suggesting that internal psychological resources serve as a critical bridge linking external environmental factors to innovation. Therefore, team leadership is willing to create an inclusive, trusting, and supportive organizational atmosphere. When teachers perceive inclusion, respect, and emotional support from leaders, they are more willing to undertake risks in their work and try new teaching methods. Simultaneously, according to Bandura’s Social Cognitive Theory, the formation of individual self-efficacy relies on vicarious experiences. University teachers often take team leaders as role models; by observing and learning from the behavioral characteristics of team leaders, they acquire vicarious experiences that enhance their innovative self-efficacy (Ren and Shen, 2024). Additionally, when teachers perceive that team leadership grants them autonomy in education and teaching, it can elevate their innovative self-efficacy, making them more willing to accept challenges in professional growth (Li et al., 2025).

Teachers’ self-efficacy exerts a positive influence on university teachers’ professional development. From the perspective of completing a social exchange, teachers’ willingness to reciprocate must be transformed into actual action, and innovative self-efficacy is precisely the psychological driving force behind this transformation. University teachers’ innovative self-efficacy manifests as a strong belief in success, believing they have the capacity to complete difficult tasks. The implementation of teacher professional development is itself a process filled with difficulties and challenges; the psychological strength provided by innovative self-efficacy helps teachers face these difficulties and challenges (Durnali and Gökbulut, 2025). In other words, when teachers feel respected and trusted by team leaders, high innovative self-efficacy encourages them to internalize professional development as a personal growth goal and shift from “being asked to develop” to “wanting to develop.” This initiative is reflected in setting personal development goals, seeking feedback, and participating in professional learning communities, among other behaviors (Al-Mahdy and Elwakil, 2026), thereby ultimately completing a positive reciprocation of leaders’ social investment. Existing research has shown that school leaders promote university teachers’ professional development precisely by influencing teachers’ internal psychological states, such as innovative self-efficacy (Gong, 2025). In sum, perceived team leadership, as an external resource input, stimulates professional development as a reciprocating behavior by enhancing teachers’ innovative self-efficacy as an internal psychological resource.

In summary, the following hypotheses are proposed:

*H2*: Perceived team leadership positively influences teachers' innovative self-efficacy.

*H3*: Innovative self-efficacy plays a mediating role in the impact of perceived team leadership on university teachers' professional development.

### 3.3 The moderating role of organizational innovation climate

Organizational innovation climate refers to an organizational factor that can be perceived by organizational members and that can influence members’ innovative behavior, including support in the form of error-tolerance mechanisms, an open collaborative culture, and innovation incentive systems (Yang et al., 2024). Social Exchange Theory holds that interactions among people are essentially processes of resource exchange based on the norm of reciprocity (Madison et al., 2025). When individuals receive positive treatment from the organization or leaders, such as support, trust, and resources, they develop a sense of obligation to reciprocate the organization and respond through positive attitudes and behaviors (Safdar et al., 2024). In this study, this theoretical framework not only explains the relationship between perceived team leadership and university teachers’ professional development, but also provides a theoretical basis for understanding the multi-path moderating mechanism of organizational innovation climate. As an important situational resource, organizational innovation climate affects the intensity of social exchange.

In recent years, the moderating role of organizational climate has received extensive attention in cross-cultural research. For example, in high power-distance cultures, organizational cultural climate can significantly moderate the influence of leadership on employee behavior (Safdar et al., 2024). Yang et al. (2024) found that organizational innovation climate moderated the mediating role of teacher agency in the relationship between digital leadership and teacher innovative behavior. These studies provide empirical support for this study’s exploration of the multiple moderating mechanisms of organizational innovation climate.

#### 3.3.1 The moderating role of organizational innovation climate in the first half of the mediation path

Social Exchange Theory suggests that individuals’ judgments of the value of external resources and their responses to those resources are highly embedded in specific organizational contexts. As a social resource input from leaders to teachers, including emotional support and trust-based empowerment, perceived team leadership is transformed into teachers’ internal psychological capital through a process that is not linear but significantly shaped by the overall organizational climate (Zhang et al., 2024). When organizational innovation climate is high, the school as a whole has formed a shared understanding that encourages exploration, tolerates failure, and allocates resources toward innovative activities. Under such conditions, teachers interpret supportive leadership behavior as a consistent organizational signal of support for innovation rather than as an occasional act by an individual leader. This perception amplifies the “perceived resource value” in the social exchange process: teachers regard the support provided by leaders not only as interpersonal trust, but also as organizationally conferred legitimacy for innovation, making them more motivated to internalize external support into the efficacy belief that “I am capable of carrying out innovative activities” (Safdar et al., 2024). By contrast, under a low organizational innovation climate, even if teachers perceive support from team leadership, they may worry about conservative organizational systems, punitive responses to innovation failure, or inadequate resource support, and therefore conclude that leadership support cannot offset the constraints imposed by the broader environment. This lowers their expected return for reciprocating leadership support and makes it difficult for external resources to be effectively converted into enhanced innovative self-efficacy. When organizational innovation climate is strong, the positive effect of perceived team leadership on innovative self-efficacy is strengthened, and the subsequent mediating effect of innovative self-efficacy on teachers’ professional development is correspondingly enhanced. When organizational innovation climate is weak, the effect of this first-half path is weakened and may even fail because of the “situational uncertainty” embedded in social exchange. This inference is also consistent with the core proposition of Social Exchange Theory that the quality of an exchange relationship depends on both parties’ shared cognition of the situation, indicating that organizational innovation climate, as a situational resource, essentially moderates the strength of the conversion from external resources to internal psychological capital by shaping teachers’ attribution of leadership support behavior.

Based on this, the study proposes:

*H4a*: organizational innovation climate positively moderates the first half of the mediation path in the model with innovative self-efficacy as the mediator.

#### 3.3.2 The moderating role of organizational innovation climate in the second half of the mediation path

Based on the core logic of Social Exchange Theory, the transformation of innovative self-efficacy into teachers’ professional development behavior is essentially the realization of “psychological resources into reciprocal behavior” within a social exchange relationship, and the extent to which this process can be achieved depends heavily on the level of support that the organizational context provides for the rules of exchange. After teachers accumulate a relatively high level of innovative self-efficacy through positive interactions with team leaders, whether they are willing to transform the belief that “I can innovate” into concrete professional development actions such as teaching reform and research exploration depends on their judgment of whether innovative behavior will receive positive feedback at the organizational level. In a context with a high organizational innovation climate, school-level error-tolerance mechanisms, innovation incentive systems, resource-allocation policies, and an open collaborative culture send teachers a clear signal: attempts at innovation will not be punished even if they fail, and successful innovative outcomes will receive organizational recognition and resource support (Afan et al., 2026). Such situational support can significantly reduce teachers’ perception of innovation risk and strengthen their expectation that investment in professional development actions will yield reasonable returns, thereby encouraging them to convert innovative self-efficacy more proactively into sustained professional development practice and complete the loop of social exchange. By contrast, under a low organizational innovation climate, even if teachers possess high innovative self-efficacy, concerns about conservative organizational evaluation, insufficient supporting resources, or the career risks of innovation failure may weaken their belief that investing in professional development can achieve reciprocal balance, thereby suppressing their willingness to convert psychological capital into actual action and causing a rupture in the social exchange chain at the “psychological resources to behavioral return” stage. This moderating effect also confirms the core proposition of Social Exchange Theory that the occurrence of exchange behavior depends not only on the reciprocal willingness of both parties, but also on the extent to which the situation safeguards the rules of exchange. It indicates that organizational innovation climate, as a situational variable, essentially moderates the strength with which innovative self-efficacy is transformed into professional development behavior by shaping teachers’ expectations of returns from innovative behavior (Vaiopoulou et al., 2026).

Based on this, the study proposes:

*H4b*: organizational innovation climate positively moderates the second half of the mediation path in the model with innovative self-efficacy as the mediator.

#### 3.3.3 The moderating role of organizational innovation climate in the direct path of the mediation model

The direct exchange relationship between perceived team leadership and university teachers’ professional development does not exist independently of organizational context; rather, its strength is systematically moderated by the shared situational cognition represented by organizational innovation climate. Social Exchange Theory suggests that individuals’ judgments of the value of leadership support behavior, the activation of reciprocity motivation, and the sustainability of exchange relationships all depend heavily on their overall cognition of the organizational environment in which they are embedded. As a collective perception reflecting the school’s innovation-supportive systems, error-tolerance mechanisms, resource allocation, and cultural consensus, organizational innovation climate essentially serves as the “situational endorsement” of the social exchange relationship between leaders and teachers and directly determines teachers’ attribution of leadership support behavior and their expectations of reciprocity (Safdar et al., 2024). When organizational innovation climate is high, the school-level shared cognition that exploration is encouraged, failure is tolerated, and innovative achievements can receive clear returns leads teachers to interpret supportive behaviors by team leaders, such as goal guidance, resource support, and emotional care, as a consistent signal of the organization’s overall support for innovation rather than as the leader’s personal preference or favor exchange. This attribution significantly amplifies the perceived value of leadership support as a social exchange resource: teachers perceive the support leaders provide not only as interpersonal trust and recognition, but also as organizationally conferred legitimacy and resource protection for professional development, thereby strengthening their reciprocal expectation that investment in professional development action will yield reasonable returns, further intensifying their motivation to reciprocate, and prompting them to transform perceived leadership support more proactively into concrete professional development behaviors such as teaching innovation and research exploration. In turn, this strengthens the direct positive effect of perceived team leadership on teachers’ professional development (Modliba et al., 2024). By contrast, under a low organizational innovation climate, even if teachers perceive supportive behavior from team leaders, concerns about conservative organizational evaluation systems, punitive risks of innovation failure, or insufficient supporting resources may lead them to interpret such support as individualized preferential treatment. As a result, they may believe that this support cannot offset the constraints of the broader environment on innovation, which lowers their expectation that the social exchange relationship is stable, weakens their motivation to reciprocate, and may even suppress their willingness to engage proactively in professional development behavior, thereby significantly weakening the direct effect of perceived team leadership on teachers’ professional development.

In the organizational context of universities, a high organizational innovation climate essentially makes the direct exchange chain between leaders and teachers function more smoothly by reinforcing the consistency between leadership support behavior and the organization’s overall innovation orientation, reducing teachers’ perception of uncertainty in exchange outcomes, and thus optimizing the “situational transaction costs” of social exchange. By contrast, a low organizational innovation climate increases the uncertainty of the exchange relationship, so that even when leaders provide support, teachers may still find it difficult to form stable expectations of reciprocity, ultimately weakening the strength of the direct path. This logic also provides a clear theoretical explanation for the boundary condition of the direct path in the moderated mediation model: as a situational variable, organizational innovation climate not only moderates the first and second halves of the mediation path, but also shapes teachers’ attribution of and expectations about leadership support, thereby moderating the direct effect of perceived team leadership on teachers’ professional development. The stronger the organizational innovation climate is, the more significant the direct positive predictive effect of perceived team leadership on teachers’ professional development becomes; the weaker it is, the less significant this effect becomes.

Based on the above logic, this study proposes:

*H4c*: organizational innovation climate positively moderates the direct path in the model with innovative self-efficacy as the mediator.

**Figure 1:**
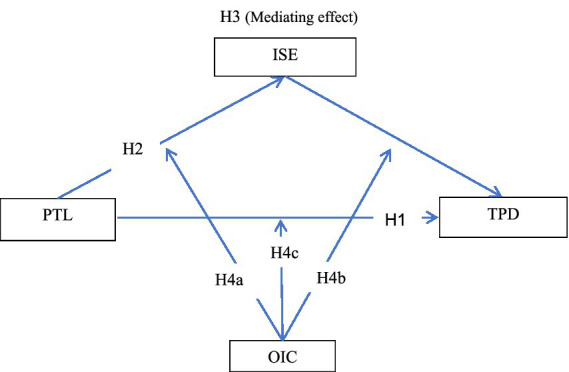
Research framework.

The theoretical model constructed in this study is shown in Figure 1.

## 4 Research design

### 4.1 Research objects

This study selected 5 local universities in the southern part of Hebei Province, China (Hengshui, Xingtai, and Handan) as the research sample. As a key node in the Beijing-Tianjin-Hebei coordinated development strategy, Hebei Province exhibits economic structures characterized by the integrated development of traditional and emerging industries. It possesses profound cultural heritage and typical regional balanced development of educational resources—both receiving the radiation effects of provincial capital cities and retaining the unique mode of local industry-education linkage. It is a microcosm of the development of local universities in northern China. Universities in this region generally face realistic challenges such as relatively limited education funding, significant pressure to optimize faculty structure, and imperfect support systems for teacher professional development (Yang and Zhou, 2025). These problems are highly consistent with the development dilemmas of similar universities nationwide, representing typical scenarios where local universities seek quality improvement under resource constraints. The 5 selected universities are all at a medium level of development within Hebei Province; they are neither key provincial construction universities nor grassroots institutions in remote areas. According to data from the Hebei Statistical Yearbook (Hebei Provincial Bureau of Statistics, 2024), such medium-development-level local universities account for approximately 50% of the total number of universities in China, and their development status accurately reflects the common characteristics of mid-tier local universities within specific regions of China. Therefore, selecting teachers from universities in this region as research objects has strong representativeness and generalizability, providing valuable and in-depth insights into the context of universities in medium-level regions.

After approval from the school ethics review committee, this study conducted a questionnaire survey of teachers from five universities using convenience sampling. In July 2024, 680 questionnaires were distributed online to university teachers. The informed consent form was prominently displayed on the first page of the questionnaire, and any questionnaire submitted without consent was excluded. After further removing questionnaires with incomplete content, logical inconsistencies in responses, excessively short completion times, or extreme values, 680 questionnaires had been recovered in total, of which 655 were valid, yielding an effective response rate of 96.3%. This study used the PROCESS macro to analyze moderated mediation. According to the power analysis of Aguinis et al. (2005), detecting a medium-sized interaction effect typically requires at least 120–150 participants, and the sample in this study fully met that requirement. Among the collected samples, 139 were male teachers (21.2%) and 516 were female teachers (78.8%); 344 held doctoral degrees (52.6%), 245 held master’s degrees (37.4%), and 66 held bachelor’s degrees or other qualifications (10.1%); 35 were professors (5.3%), 139 were associate professors (21.2%), 413 were lecturers (63.1%), and 68 were teaching assistants (10.4%).

In interpreting the influencing factors of university teachers’ professional development, this article places greater emphasis on teachers’ own perceptions of professional development, and the measurement of team leadership was obtained through teachers’ perceptions; therefore, a certain degree of subjectivity may exist, lacking multi-dimensional measurement assessment and comparison.

### 4.2 Research tools

#### 4.2.1 Perceived team leadership scale

To measure perceived team leadership, this study adopted an adapted version of the Team Leadership Scale to assess university teachers’ perceptions of team leadership. The original version of the scale was developed by Morgeson et al. (2010). To ensure a high degree of fit between the instrument and the research context and objectives, the research team carried out a localized revision while retaining the core constructs of the original scale. The revision process included the following steps: first, several researchers other than the first author independently reviewed the comprehensiveness and representativeness of the questionnaire items; second, redundant content was removed and semantically repetitive items were merged through focused discussion.

For example, in the “Define Mission” dimension, Item 1 and Item 3 were merged because aspects and goals overlapped. The “Train and Develop Team” dimension was reduced from 5 items to 3; original Item 2 (“Team helps new members learn how to do the work”) and Item 4 (“Team helps new members further improve skills”) were merged. Item 3 (“Team provides task-related guidance”) and Item 5 (“Team helps members learn lessons from past events or experiences”) were also merged. This consolidation aimed to streamline the questionnaire and reduce redundancy. Consequently, perceived team leadership was measured using 6 dimensions: “Define Mission,” “Train and Develop Team,” “Provide Feedback,” “Solve Problems,” “Provide Resources,” and “Support Social Climate.” Each dimension contained three items. A total of 18 items.

The scale utilized a 5-point Likert scoring system (1 = Very Non-conforming, 5 = Very Conforming). Higher scores indicate that respondents perceive stronger leadership in that dimension. In this study, the Cronbach’s Alpha coefficients for the subscales were.845, 0.825, 0.859, 0.836, 0.832, and 0.868, respectively. The total Cronbach’s Alpha coefficient was 0.911, all greater than 0.7, indicating good internal stability and consistency (see Table 1).

**Table 1:** Reliability analysis of the perceived team leadership scale.

Name of scale	Dimension	Dimension reliability	Scale reliability
Perceived team leadership	Define mission	0.845	0.911
	Train and develop team	0.825	
	Provide feedback	0.859	
	Solve problems	0.836	
	Provide resources	0.832	
	Support social climate	0.868	

The confirmatory factor analysis results indicated that the measurement model fit the data well: *χ*^2^/*df* = 2.359, below the recommended critical value of 5.00; the Goodness-of-Fit Index (GFI) = 0.954, Incremental Fit Index (IFI) = 0.972, Normed Fit Index (NFI) = 0.952, Comparative Fit Index (CFI) = 0.972, and Tucker-Lewis Index (TLI) = 0.966, all exceeding the recommended threshold of 0.900; and the Root Mean Square Error of Approximation (RMSEA) = 0.046 and Standardized Root Mean Square Residual (SRMR) = 0.0309, both below the acceptable threshold of 0.080 (Schumacker and Lomax, 2004; Hair et al., 1998). All fit indices reached good levels, indicating that the questionnaire had good structural validity (see Table 2).

**Table 2:** Fit indices for the perceived team leadership model.

Model	χ^2^/*df*	GFI	IFI	NFI	CFI	TLI	RMSEA	SRMR
	2.359	0.954	0.972	0.952	0.972	0.966	0.046	0.0309

#### 4.2.2 Teacher professional development scale

For the measurement of teacher professional development, this study adopted the “Teacher Professional Development Scale” developed by García-Martínez et al. (2019). It includes four constructs: “Organize and Promote Situations,” “Teamwork,” “Use of New Technologies,” and “Organize One’s Own Continuous Training.”

To ensure the applicability and accuracy of the scale in the context of this study, the original questionnaire of 43 items was adapted into 27 items. The original questionnaire of 43 items was adapted and subjected to expert content validity review, resulting in a 27-item questionnaire. For instance, original Item 5 in “Organize and Promote Situations” (“Teachers should maintain certain daily teaching routines while also making timely changes and innovations”) highly overlapped with Item 4 (“Must constantly innovate in the classroom”). We retained the more direct “innovation” expression and deleted the composite expression of “routine” and “change” to make the construct more accurate and concise. In “Teamwork,” original Item 6 (“Understanding school management functions is important and necessary”) was deleted as it related to cognition of administrative management, which has a weak association with the core construct of professional interaction among teachers. In “Use of New Technologies,” specific software skill items (Original 6/7/9, e.g., proficiency in Word, Excel, Email) were deleted as these are basic digital literacies and default skills in the current era, insufficient to distinguish professional development levels. The last construct, “Organize One’s Own Continuous Training,” had reverse items removed. The final scale comprised 27 items using 5-point Likert scoring (1 = Strongly Disagree, 5 = Strongly Agree). Higher scores indicate higher levels of teachers’ professional development.

The Cronbach’s Alpha coefficients for the four constructs were 0.917, 0.881, 0.859, and 0.897, respectively. The total Cronbach’s Alpha was 0.935, indicating good reliability (see Table 3).

**Table 3:** Reliability analysis of the teacher professional development scale.

Name of scale	Dimension	Dimension reliability	Scale reliability
Teacher professional development	Organize and promote situations	0.917	0.935
	Teamwork	0.881	
	Use of new technologies	0.859	
	Organize one’s own continuous training	0.897	

The confirmatory factor analysis results indicated that the measurement model fit the data well: *χ*^2^/df = 1.555, below the recommended critical value of 5.00; the Goodness-of-Fit Index (GFI) = 0.950, Incremental Fit Index (IFI) = 0.981, Normed Fit Index (NFI) = 0.948, Comparative Fit Index (CFI) = 0.985, and Tucker-Lewis Index (TLI) = 0.979, all exceeding the recommended threshold of 0.900; and the Root Mean Square Error of Approximation (RMSEA) = 0.029 and Standardized Root Mean Square Residual (SRMR) = 0.0295, both below the acceptable threshold of 0.080 (Schumacker and Lomax, 2004; Hair et al., 1998). All fit indices reached good levels, indicating that the questionnaire had good structural validity (see Table 4).

**Table 4:** Fit indices for the teacher professional development model.

Model	χ^2^/*df*	GFI	IFI	NFI	CFI	TLI	RMSEA	SRMR
	1.555	0.950	0.981	0.948	0.985	0.979	0.029	0.0295

#### 4.2.3 Innovative self-efficacy scale

Innovative self-efficacy was measured using the unidimensional scale developed by Tierney and Farmer (2002), this scale has good reliability and validity and is suitable for the participants in this study. It contains four items. Typical items include “I think I am good at proposing novel ideas” and “I am confident in my ability to solve problems creatively,” among others. The scale consists of 4 items with 5-point Likert scoring (1 = Very Disagree, 5 = Very Agree). Reliability analysis yielded a Cronbach’s Alpha = 0.870, indicating good reliability. CFA results indicated a saturated model (*χ*^2^/df = 0, df = 0), forming a perfect fit with the data (Wu, 2010). All factor loadings reached significance (*p* < 0.001), with standardized factor loadings ranging between.705 and.836, all greater than.500. The Composite Reliability (CR) of the latent variable was 0.844 (>0.700), and the Average Variance Extracted (AVE) was 0.576 (>0.500) (Fornell and Larcker, 1981), indicating good convergent validity.

#### 4.2.4 Organizational innovation climate scale

Organizational innovation climate was measured using the “Organizational Innovation Climate Scale” developed by Zheng et al. (2009). This scale has good reliability and validity and is suitable for the participants in this study. It includes seven constructs: “Incentive Mechanism,” “Leadership Practice,” “Team Effort,” “Superior Support,” “Resource Guarantee,” “Organizational Promotion,” and “Autonomous Work,” totaling 21 items. Typical items include “The school’s reward system makes everyone enthusiastic about innovation” and “My leader often carries out work creatively,” among others. The scale used 5-point Likert scoring (1 = Very Non-conforming, 5 = Very Conforming). The Cronbach’s Alpha coefficients for the dimensions were.850, 0.791, 0.790, 0.788, 0.838, 0.756, and 0.790, respectively. The total Cronbach’s Alpha was 0.918, indicating good reliability (see Table 5).

**Table 5:** Reliability analysis of the organizational innovation climate scale.

Name of scale	Dimension	Dimension reliability	Scale reliability
Organizational innovation climate	Incentive mechanism	0.850	0.918
	Leadership by example	0.791	
	Team collaboration	0.790	
	Superior support	0.788	
	Resource guarantee	0.838	
	Organizational promotion	0.756	
	Autonomous work	0.790	

The confirmatory factor analysis results indicated that the measurement model fit the data well: *χ*^2^/df = 2.054, below the recommended critical value of 5.00; the Goodness-of-Fit Index (GFI) = 0.950, Incremental Fit Index (IFI) = 0.969, Normed Fit Index (NFI) = 0.941, Comparative Fit Index (CFI) = 0.969, and Tucker-Lewis Index (TLI) = 0.964, all exceeding the recommended threshold of 0.900; and the Root Mean Square Error of Approximation (RMSEA) = 0.040 and Standardized Root Mean Square Residual (SRMR) = 0.0296, both below the acceptable threshold of 0.080 (Schumacker and Lomax, 2004; Hair et al., 1998). All fit indices reached good levels, indicating that the questionnaire had good structural validity (see Table 6).

**Table 6:** Fit indices for the organizational innovation climate model.

Model	χ^2^/*df*	GFI	IFI	NFI	CFI	TLI	RMSEA	SRMR
	*2*.054	0.950	0.969	0.941	0.969	0.964	0.040	0.0296

### 4.3 Data analysis

After standardizing all variables, the moderated mediation model was tested using the SPSS PROCESS Macro provided by Hayes (2017). Bootstrap-based 95% confidence intervals generated from 5,000 random samples were used to test the indirect effects. Specifically, to examine whether innovative self-efficacy mediated the relationship between perceived team leadership and university teachers’ professional development, we first used PROCESS Macro (Model 4). We then used PROCESS Macro (Model 59) to test whether organizational innovation climate moderated the mediation effect.

## 5 Research results

### 5.1 Common method bias test

Since all key variables in each questionnaire were answered by the same subject, a common source bias issue might exist. To avoid this, an anonymous measurement method was employed during the survey. Additionally, Harman’s single-factor test was used. All items of the four main variables were subjected to unrotated principal component factor analysis. Results showed KMO = 0.926 (>0.800), and Bartlett’s test of sphericity was significant (*p* = 0.001). Eighteen factors with eigenvalues greater than 1 were extracted, and the first principal component explained 23.197% of the variance, which is less than the critical standard of 40%. This indicates that common method bias does not significantly affect the relationships between variables in this study.

In addition to Harman’s single-factor test, several procedural remedies were applied to reduce the potential impact of common method bias. Respondents were assured of anonymity and confidentiality, and informed that there were no right or wrong answers to minimize evaluation apprehension. Furthermore, the questionnaire items were carefully worded and arranged to reduce respondents’ tendency to infer relationships among variables. These procedural controls are consistent with prior methodological recommendations and help mitigate concerns regarding common method variance.

### 5.2 Correlation analysis

Pearson correlation analysis was conducted for all variables. As shown in Table 7, correlation coefficients ranged between.169 and.585, and all correlations reached positive significance (*p* < 0.001). Moderate to low correlations existed between variables, and no coefficient exceeded.800, indicating no collinearity issues (Maruyama, 1998).

**Table 7:** Correlation analysis of variables (*n* = 655).

Variable	Mean	SD	Perceived team leadership	Teacher professional development	Innovative self-efficacy	Organizational innovation climate
Perceived team leadership	3.787	0.630	**1**			
Teacher professional development	2.990	0.513	0.528***	**1**		
Innovative self-efficacy	5.049	1.046	0.397***	0.585***	**1**	
Organizational innovation climate	3.474	0.558	0.169***	0.352***	0.401***	**1**

*p < 0.05, **p < 0.01, ***p < 0.001. Values on the diagonal represent the autocorrelation coefficients of the variables.

### 5.3 Regression analysis

After validity and reliability testing and correlation analysis, models were constructed with Perceived Team Leadership as the independent variable, Innovative Self-Efficacy as the mediator, and Teacher Professional Development as the dependent variable. Model 1 validated H1, Model 2 validated H2, and Model 3 combined with Model 1 validated H3. Results showed: Perceived Team Leadership had a significant positive predictive effect on Teacher Professional Development (*β* = 0.430, *p* < 0.001), and on Innovative Self-Efficacy (*β* = 0.659, *p* < 0.001). Hypotheses H1 and H2 were supported (see Table 2). As shown in Table 8.

**Table 8:** Test of mediation effect of innovative self-efficacy.

Predictor	Model 1: teacher professional development		Model 2: innovative self-efficacy		Model 3: teacher professional development	
	*β*	*t*	*β*	*t*	*β*	*t*
Perceived team leadership	0.430	11.039***	0.659	11.057***	0.351	11.039***
Innovative self-efficacy	–	–	–	–	0.219	14.055***
*R* ^2^	0.279		0.158		0.446	
*F*	252.074***		122.252***		122.566***	

*p < 0.05, **p < 0.01, ***p < 0.001.

Using Model 4 in the SPSS macro program PROCESS 4.1 for mediation testing, Model 3 results compared to Model 1 showed that after adding the Innovative Self-Efficacy variable, the direct effect of Perceived Team Leadership on Teacher Professional Development decreased but remained significant (*β* = 0.351, *p* < 0.001). Thus, the partial mediation effect of Innovative Self-Efficacy was established, accounting for 30.0% of the total effect. Hypothesis H3 was verified.

### 5.4 Test of moderated mediation effect

After standardization, Model 59 in the SPSS plugin PROCESS provided by Hayes was used to test the moderated mediation. Results are shown in Table 9: The interaction of Perceived Team Leadership X Organizational Innovation Climate significantly positively predicted Teacher Professional Development (*β* = 0.250, *p* < 0.001) and Innovative Self-Efficacy (*β* = 0.657, *p* < 0.001). The interaction of Innovative Self-Efficacy and Organizational Innovation Climate on Teacher Professional Development was also significant (*β* = 0.054, *p* < 0.05, 95% confidence interval of 5,000 bootstrap samples did not contain 0). Hypotheses H4a-c were supported. Organizational Innovation Climate positively moderated all paths of the mediation framework (see Table 10).

**Table 9:** Test of moderated mediation model.

Predictors	Model 2 (teacher professional development)			Model 1 (innovative self-efficacy)		
	*B(SE)*	*t*	95%CI	*B(SE)*	*t*	95%CI
Perceived team leadership	0.307 (0.026)	11.592***	[0.255, 0.359]	0.614 (0.054)	11.270***	[0.507, 0.721]
Innovative self-efficacy	0.168 (0.017)	9.826***	[0.135, 0.202]			
Organizational innovation climate	0.147 (0.027)	5.433***	[0.094, 0.201]	0.644 (0.059)	10.920***	[0.528, 0.760]
Team leadership X Org. Innovation Climate	0.250 (0.041)	6.071***	[0.169, 0.331]	0.657 (0.088)	7.396***	[0.482, 0.831]
Innovative self-efficacy X Org. innovation climate	0.054(0.027)	1.973*	[0.003, 0.108]			
*R* ^2^	0.503			0.328		
*F*	131.427***			106.304***		

Standard Deviation: 95% CI, bootstrapped confidence interval.

*p < 0.05, **p < 0.01, ***p < 0.001.

**Table 10:** Test of moderating effects.

Organizational innovation climate level	Effect value	SE	*p*-value	95% confidence interval
Low (*Z*^2^ = −0.664)	0.023	0.012	<0.001	[0.002, 0.050]
Medium (*Z*^2^ = 0.049)	0.111	0.015	<0.001	[0.082, 0.143]
High (*Z*^2^ = 0.621)	0.207	0.033	<0.001	[0.146, 0.274]

**Figure 2:**
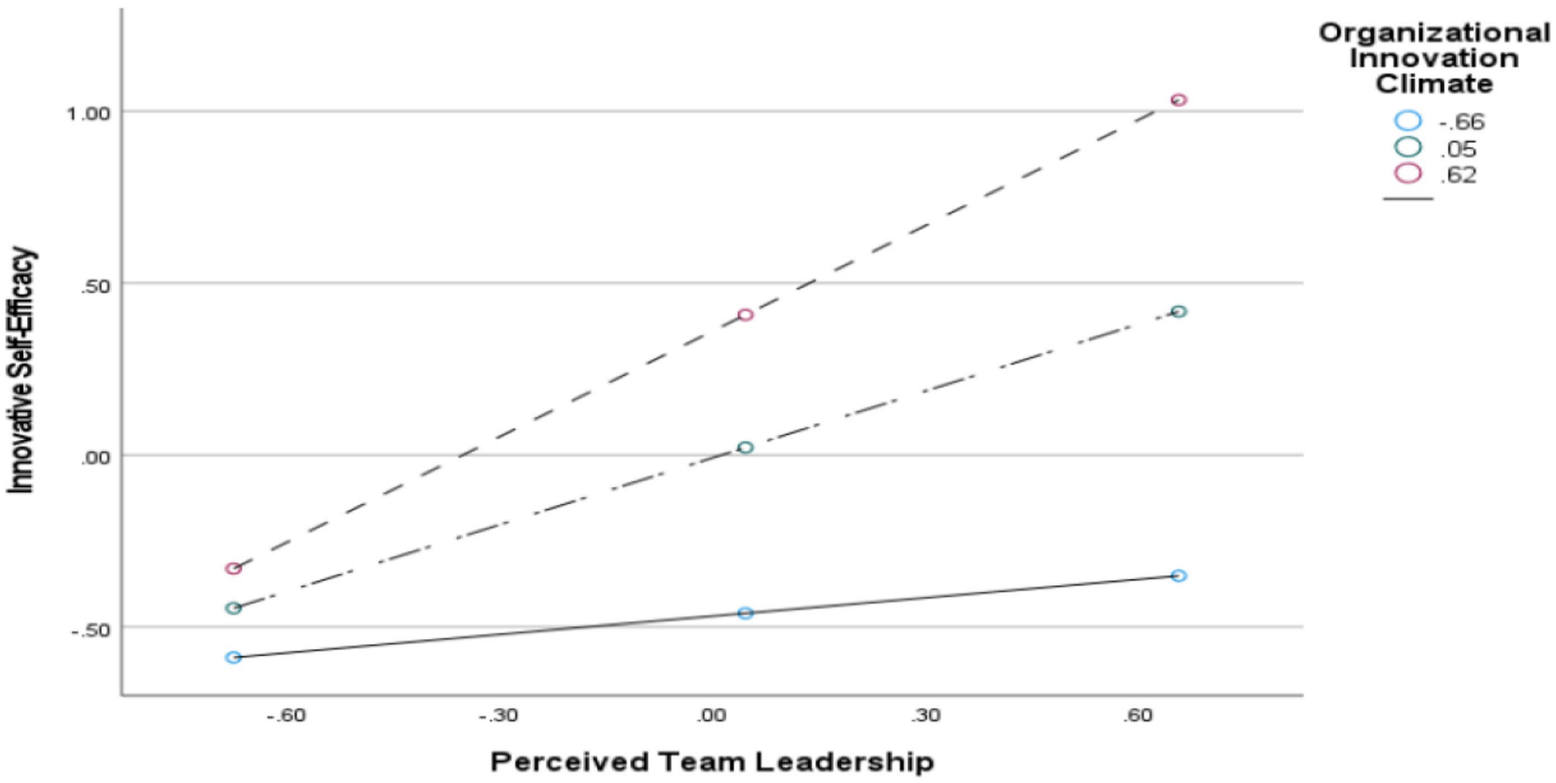
The moderating role of organizational innovation climate in the relationship between innovative self-efficacy and perceived team leadership.

**Figure 3:**
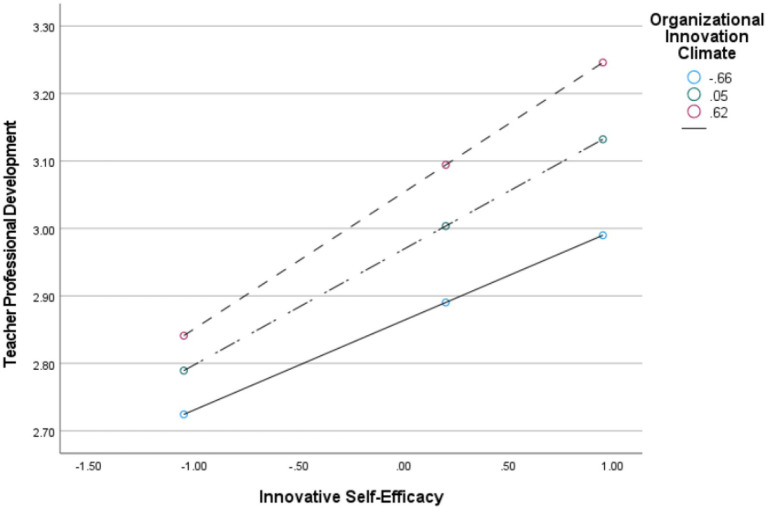
The moderating role of organizational innovation climate in the relationship between university teachers’ professional development and innovative self-efficacy.

**Figure 4:**
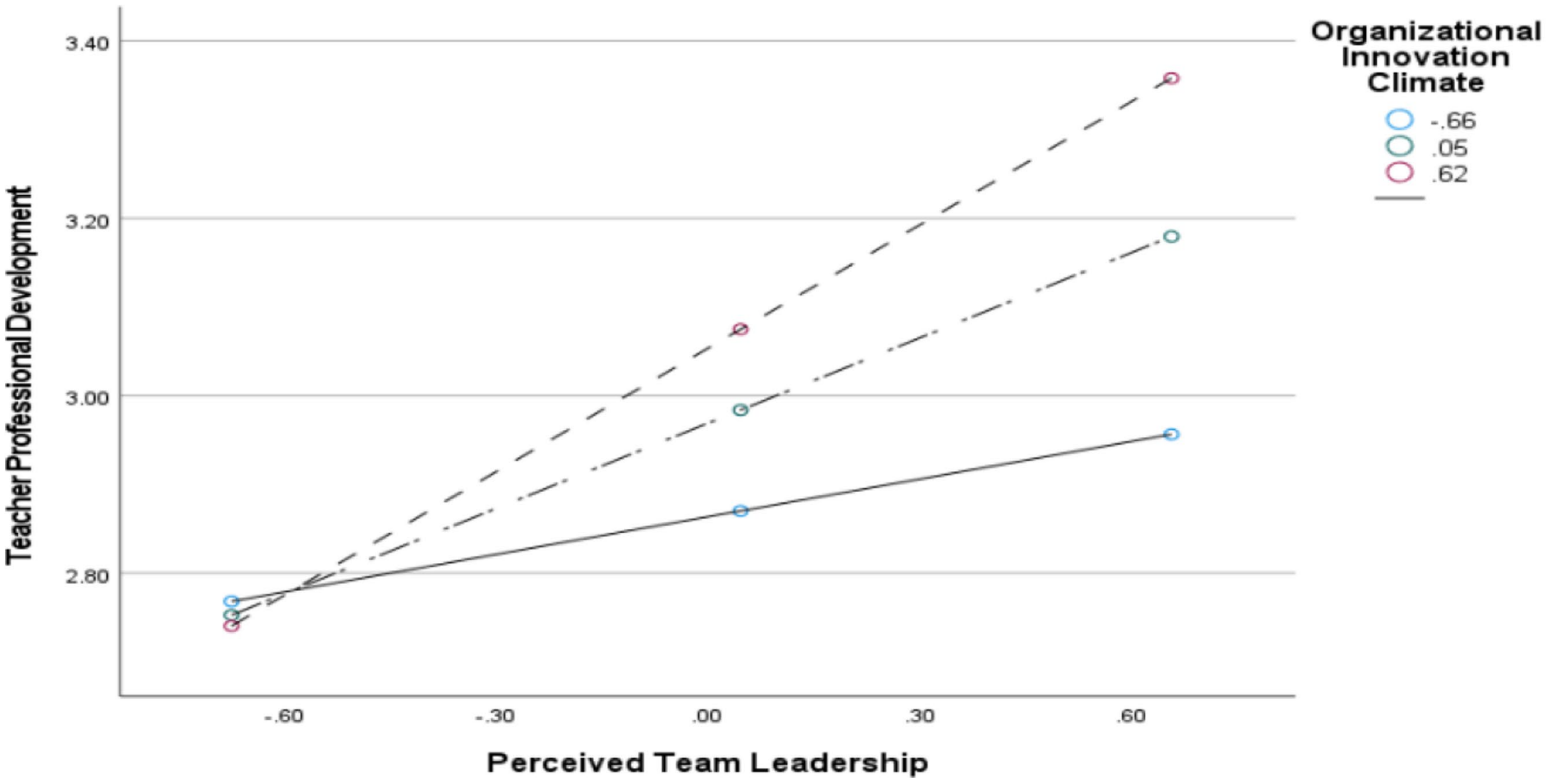
The moderating role of organizational innovation climate in the relationship between university teachers’ professional development and perceived team leadership.

To further verify the moderating effect, simple slope analysis was conducted using the standardized score of Organizational Innovation Climate plus or minus one standard deviation (results depicted in Figures 2–4 and Table 10). When Organizational Innovation Climate is stronger, the positive relationships between other variables (e.g., Perceived Team Leadership → Innovative Self-Efficacy, or Perceived Team Leadership/Innovative Self-Efficacy → Professional Development) become more significant and intense. Briefly, under a strong organizational innovation climate, teachers more easily perceive leadership support and convert it into stronger innovation confidence, which is then more effectively translated into actual action. Conversely, if the atmosphere is suppressed, even if leadership supports innovation, teachers may lack confidence due to environmental limitations, or they may fear failure or lack resources, preventing the conversion of confidence into practice. For universities with low Organizational Innovation Climate, the direct effect of Perceived Team Leadership on Teacher Professional Development was relatively strong (Effect = 0.236, 95% CI [0.0021, 0.0503], not containing 0). For universities with high Organizational Innovation Climate, the direct effect was 0.207 (95% CI [0.1465, 0.2743], not containing 0). Furthermore, from the analysis of Figures 2–4, it can be seen that organizational innovation climate not only strengthened the influence of perceived team leadership on teacher professional development, but also enhanced the influence of perceived team leadership on innovative self-efficacy. However, the moderating effect on the relationship between innovative self-efficacy and teacher professional development was notably weaker.

## 6 Discussion

### 6.1 The direct effect of perceived team leadership on teacher professional development

Based on the reciprocity principle of Social Exchange Theory, this study empirically examined the positive effect of perceived team leadership on university teachers’ professional development. The results confirmed that university teachers’ positive perception of team leadership can effectively enhance their level of professional development, providing new empirical evidence for applying the reciprocity principle in the organizational context of higher education institutions. This conclusion aligns with previous studies (Bao, 2018; Comstock et al., 2021). While prior research mainly discussed specific leadership styles without interpreting from the perspective of team leadership—especially leadership perceived by teachers—this study starts from perceived team leadership, further verifying its important status in the process of teachers’ professional growth. Results indicate that the higher the perceived team leadership, the better the professional development of university teachers. In other words, when university teachers have a high perception of team leadership, they believe that leaders can clearly provide goal guidance, effectively coordinate internal and external resources, and lead by example. This positive perception allows teachers to experience a stronger supportive atmosphere, thereby stimulating intrinsic motivation for professional development and promoting continuous growth in teaching, research, and academic cooperation (Mubarak et al., 2025).

When university teachers have a high perception of team leadership, they believe that team leaders can clearly formulate development goals and visions for the entire team; this positive perception of goal guidance helps teachers integrate personal career goals with the development direction of the team, which aids in enhancing teachers’ professional development (Khan et al., 2020; Ma, 2011). The stronger the perception of team leadership’s ability to coordinate resources, the more teachers believe that team leaders can actively secure external resources (such as project funding and cooperation opportunities) for them. This perception of resource support provides necessary conditions for teachers to conduct research and teaching, thereby promoting professional development (Liu et al., 2018). When teachers perceive an open, inclusive, and trusting team atmosphere, this positive perception encourages them to engage in academic research and teaching reform, also promoting professional development (Wang and Nie, 2021). These findings further refine Social Exchange Theory: perceived leadership can drive professional development because it simultaneously provides instrumental resources and emotional resources, both of which jointly constitute the source of teachers’ motivation to reciprocate.

In summary, this study further emphasizes the importance of perceived team leadership within organizations. Organizations should strengthen the cultivation of team leadership and foster a positive team climate; only in this way can the level of members’ perception of team leadership within the organization be enhanced.

### 6.2 The partial mediating role of innovative self-efficacy

The results show that university teachers’ innovative self-efficacy has a mediating effect between perceived team leadership and professional development. This result is consistent with previous research views (Comstock et al., 2021). It indicates that perceived team leadership not only has a direct impact on university teachers’ professional development but also indirectly promotes it by enhancing the internal psychological mechanism of innovative self-efficacy. That is, the stronger the perceived team leadership, the more it can effectively enhance university teachers’ confidence and belief in their own innovation capabilities, i.e., enhancing their “innovative self-efficacy.” When teachers believe they have the ability to propose novel teaching methods, conduct original research, and solve complex academic problems, they are more likely to actively seek professional development opportunities.

According to Social Exchange Theory, interactions between individuals and organizations or leaders are based on reciprocity, trust, and resource exchange. When individuals perceive support from the organization or leader, they generate an intrinsic sense of obligation to repay and positive mentalities, thereby demonstrating positive behaviors. That is, the support of team leadership perceived by teachers encourages them to bravely explore directions for innovation and development, stimulating the formation of innovative self-efficacy, which further stimulates teachers’ professional development (Mubarak et al., 2021; Schunk and DiBenedetto, 2020). From the perspective of the work environment, when teachers feel a loose and harmonious atmosphere provided by the organization, they feel supported. Out of repayment, teachers are more willing to unleash their potential and demonstrate more innovative emotions and behaviors, thereby promoting their own career growth (Zhang et al., 2015). From the aspect of teacher cooperation, when teachers receive emotional support from leaders during cooperation, they perceive collective investment and trust, stimulating a sense of responsibility to repay the team. This responsibility prompts teachers to proactively improve their problem-solving abilities, gradually building confidence in innovative behaviors, thereby promoting their professional growth (Van Dijk et al., 2020). Team leadership encouraging learning and training constitutes an input of organizational resources; after receiving resources, teachers generate a psychological obligation to repay, enabling them to quickly master professional knowledge and skills, enhancing self-confidence, and elevating professional development levels (Zhang et al., 2015). These findings indicate that within Social Exchange Theory, the exchange of socio-emotional resources not only occurs in interpersonal interaction, but can also be internalized into individuals’ psychological capital, thereby driving higher-level reciprocating behavior.

Therefore, innovative self-efficacy occupies a very important position in individuals’ professional development. To enhance individuals’ innovative self-efficacy, organizations should focus on creating a supportive cooperative atmosphere, building incentive and support systems, and strengthening personal internal beliefs and capabilities.

### 6.3 The moderating role of organizational innovation climate in the mediation framework

The moderated mediation model validated in this study indicates that organizational innovation climate exerts significant positive moderating effects on the first half of the path, the second half of the path, and the direct path within the chain of “perceived team leadership → innovative self-efficacy → teachers’ professional development.” This finding not only echoes the core proposition of the “situation-individual-behavior” interaction, but also provides new empirical support for existing conclusions regarding the boundary role of organizational innovation climate. Regarding the first half of the mediation path, the simple slope analysis showed that when organizational innovation climate was one standard deviation above the mean, the positive predictive coefficient of perceived team leadership on innovative self-efficacy was 0.657 (*p* < 0.001); when organizational innovation climate was one standard deviation below the mean, the coefficient declined to 0.321 (*p* < 0.05), and the difference between the two coefficients was significant. This result is highly consistent with Yang et al.’s (2024) finding that organizational innovation climate strengthens the transformation of external leadership factors into individuals’ psychological capital, and it also confirms Xu et al.’s (2022) theoretical view that the quality of social exchange relationships depends on situational support. The internal mechanism is that, under a high innovation climate, teachers interpret leaders’ trust-based empowerment and resource support as a consistent organizational signal supporting innovation rather than as the leader’s personal random preference. This cognition amplifies the perceived resource value of leadership support and strengthens the efficiency with which external resources are converted into innovative self-efficacy (Zhang et al., 2024). Under a low innovation climate, by contrast, teachers’ concerns about conservative organizational institutions and innovation risks reduce their expectations of reciprocity, making it difficult for external support to be effectively internalized into psychological capital. This also explains why some universities, despite strong support from team leaders, still generally face the practical dilemma of insufficient teacher confidence in innovation.

Regarding the second half of the mediation path, the test of the moderating effect showed that the interaction term between organizational innovation climate and innovative self-efficacy had a predictive coefficient of 0.054 (*p* < 0.05) for teachers’ professional development. Although the effect size was weaker than that of the first half of the path, it was still significant, supplementing current understanding of the boundary conditions under which psychological capital is transformed into behavior. Compared with previous studies, Safdar et al. (2024) found in cross-cultural leadership research that the strength of the moderating effect of organizational climate on the transformation of psychological resources into behavior is influenced by cultural factors. The findings of the present study further indicate that, in the context of universities as knowledge-based organizations, even when teachers have high innovative self-efficacy, its conversion into professional development action still requires situational support at the organizational level. Specifically, under a high innovation climate, school-level error-tolerance mechanisms, innovation incentive systems, and resource allocation policies can reduce teachers’ perception of innovation risk and strengthen their expectation that investment in professional development can bring reasonable returns, thereby promoting the efficient conversion of innovative self-efficacy into concrete actions such as teaching reform and research exploration (Afan et al., 2026). Under a low innovation climate, however, even if teachers believe they are capable of carrying out innovative activities, concerns about career risks after failure, insufficient resources, or unfair evaluation may suppress their willingness to act, causing a rupture in the social exchange chain at the stage of “psychological capital → behavioral return.” It is worth noting that this study found the moderating effect in the second half of the path to be weaker than that in the first half, which is consistent with the theoretical expectation that once individual beliefs are formed, they tend to possess relative stability. This suggests that when individuals are convinced of their own innovative capacity, they may still proactively seek resources to achieve their goals even when the external environment imposes certain constraints. This also provides a theoretical basis for some universities to promote professional development by enhancing teachers’ innovative self-efficacy under conditions of limited resources.

Regarding the direct path (perceived team leadership → teachers’ professional development), the results showed that when organizational innovation climate was one standard deviation above the mean, the direct effect of perceived team leadership on teachers’ professional development was 0.207 [95% CI = (0.146, 0.274)]; when organizational innovation climate was one standard deviation below the mean, the direct effect declined to 0.023 [95% CI = (0.002, 0.050)]. The difference between the two effects was significant, extending current leadership research on the boundary conditions of direct effects. Compared with previous studies, Kilag and Sasan (2023) only verified the direct effect of instructional leadership on teachers’ professional development, whereas the present study further reveals that the strength of this direct effect depends heavily on the support of organizational innovation climate: under a high innovation climate, teachers regard leaders’ supportive behavior as part of the organization’s overall innovation orientation, which amplifies perceived resource value and reciprocity motivation and thereby strengthens the direct path (Modliba et al., 2024); under a low innovation climate, leadership support is interpreted as individualized preferential treatment, which cannot offset the inhibitory effect of environmental constraints on reciprocity motivation and thus significantly weakens the direct effect. This finding confirms the core logic of Social Exchange Theory: the direct exchange relationship between leaders and teachers does not exist independently of organizational context; rather, organizational innovation climate essentially constitutes the “situational endorsement” of the exchange relationship and directly determines teachers’ attribution of leadership support behavior and their expectations of reciprocity (Safdar et al., 2024). Taken together, the moderating effects along the three paths show that organizational innovation climate is not only an “amplifier” of leadership effectiveness, but also the “fundamental condition” for the effective operation of the entire mediation mechanism. Only when team leadership is aligned with a strong organizational innovation climate can the endogenous motivation for teachers’ professional development be maximally activated. This provides clear empirical evidence for university administrators to formulate teacher development policies from both the dimensions of leadership enhancement and climate cultivation.

Although the findings are primarily interpreted through the lens of Social Exchange Theory, alternative explanations merit consideration. For example, university teachers’ professional development may also be influenced by factors such as access to institutional resources or intrinsic career motivation, rather than perceived leadership alone. In addition, reverse causality cannot be entirely ruled out, as teachers with higher levels of professional development may be more inclined to evaluate team leadership positively. Nevertheless, the theoretical framework adopted in this study conceptualizes perceived team leadership as a contextual social resource that precedes psychological empowerment, which is consistent with prior theory-driven research. Future studies employing longitudinal or experimental designs could further examine these alternative explanations.

## 7 Conclusions and suggestions

### 7.1 Conclusion

This study provides empirical support for a theoretically grounded model linking perceived team leadership, innovative self-efficacy, and university teachers’ professional development within the context of Chinese higher education. By applying Social Exchange Theory to this setting, the findings validate and contextualize established leadership and psychological mechanisms rather than introducing a new theoretical framework. Accordingly, the study contributes context-specific evidence that strengthens and refines existing theoretical understandings of leadership-driven professional development.

### 7.2 Theoretical significance

This study adopts Social Exchange Theory to research university teachers’ professional development, constructing a theoretical model centered on relational reciprocity. It reveals the social exchange mechanism between perceived team leadership and university teachers based on trust and repayment obligation, emphasizing the important roles of interactivity, affectivity, and obligation in university organizational contexts, enriching the interpretation of leadership effectiveness in the field of educational management.

This study validates the psychological bridging role of innovative self-efficacy as a mediator in the process of perceived team leadership influencing university teachers’ professional development. Existing research mostly focuses on the impact of external incentives on teacher behavior, paying less attention to the mediating mechanism of individual psychological resources. By introducing innovative self-efficacy, this study reveals how teachers’ perception of leadership support enhances their innovation beliefs, thereby stimulating their intrinsic motivation to actively participate in teaching reform and scientific research innovation.

This study introduces organizational innovation climate as a moderating variable, emphasizing the “amplifier” function of organizational cultural context in the social exchange process. The research finds that the effect of teachers’ perception of team leadership does not exist in isolation but is integrated into the organizational atmosphere. High organizational innovation climate can reinforce the connection between perceived team leadership and university teachers’ professional development, promoting the transformation of social exchange relationships into positive behaviors, while low organizational innovation climate may inhibit this process. This discovery echoes the interaction of “Context-Individual-Behavior” in organizational behavior research and promotes research on university teachers’ professional development.

### 7.3 Practical implications

First, systematically enhance team leadership. Schools should conduct specialized training for team leaders, focusing on strengthening their comprehensive capabilities in goal guidance, resource coordination, communication support, and emotional care. Leaders should be guided to establish high-quality social exchange relationships through behaviors such as respect, trust, and care, thereby effectively stimulating the intrinsic development motivation of university teachers.

Second, focus on stimulating teachers’ innovative self-efficacy. Managers can help university teachers establish small plans or goals one by one; by achieving small goals, teachers’ confidence in self-innovation is increased. Excessive pressure should be avoided to enhance teachers’ confidence in facing teaching and research challenges.

Finally, foster a positive organizational innovation climate. Universities should establish an inclusive, open, and trusting culture. Creating an organizational atmosphere that supports innovation, encourages exploration, and tolerates failure can effectively enhance university teachers’ sense of trust and belonging to the organization, enabling the role of team leadership to be amplified, thereby promoting teacher professional development.

### 7.4 Limitations and future directions

Although this study provides certain theoretical and practical insights, some limitations need to be addressed. An important limitation of this study lies in the severely imbalanced gender distribution of the sample, with a higher proportion of female respondents. Given that existing research suggests gender may influence how individuals perceive leadership behaviors and the formation mechanisms of innovative self-efficacy, the current results may more closely reflect the experiential patterns of female teachers. Therefore, in future research, gender could be incorporated into the analytical framework as a moderating variable. Additionally, multiple sampling methods could be employed during sampling to ensure a more balanced gender ratio, thereby enhancing the external validity of research findings. Furthermore, future research could test the model of this study in more diverse higher education contexts (such as “Double First-Class” universities, vocational colleges, and institutions with significant regional or disciplinary structural differences) to progressively establish stronger external validity and theoretical generalizability.

Regarding the adaptation of scales measuring team leadership and teacher professional development, it should be acknowledged that external expert review or independent pilot testing was not conducted. If future research could incorporate domain expert review or conduct small-scale pilot testing, it would further enhance the reliability and validity of the scales. This experience also reminds us to strengthen the rigor and transparency of methodological procedures in subsequent work.

Due to constraints in research time and resources, this article used cross-sectional data. The inference of causal relationships between variables and the test of influence mechanisms are less persuasive compared to longitudinal studies. The results make it difficult to reflect the dynamic evolution laws among perceived team leadership, innovative self-efficacy, organizational innovation climate, and teacher professional development. For example, whether individuals’ professional development would subsequently enhance positive perceptions of leadership, and whether organizational climate and self-efficacy mutually influence and reinforce each other over the long term. Therefore, future research needs to strengthen longitudinal tracking studies to explore the causal mechanisms between perceived team leadership and university teachers’ professional development, improving the persuasiveness and applicability of the theoretical model.

Furthermore, in interpreting the influencing factors of university teachers’ professional development, the article discussed the impact of teachers’ individuals on professional development, focusing more on teachers’ own perceptions. The measurement of team leadership was obtained through teachers’ perceptions, so there may be a certain degree of subjectivity, lacking multi-dimensional measurement assessment and comparison. Therefore, future research can explore the impact of team leadership and various styles of leaders on university teachers’ professional development, and can also consider combining school, teacher, and individual factors, applying cross-level analysis methods to analyze and discuss factors influencing university teachers’ professional development.

## Data Availability

The raw data supporting the conclusions of this article will be made available by the authors, without undue reservation.

## References

[ref1] Afan A. Achmad G. N. Adhimursandi D. (2026). Knowledge sharing and organizational support: keys to lecturers' creative self-efficacy and innovation. Indonesian Interdiscip. J. Sharia Econ. 9, 26–42. doi: 10.31538/iijse.v9i1.7521

[ref2] Afrianty T. W. Utami H. N. Sasmita E. E. Putra F. R. R. Wulandari T. D. (2025). Linking servant leadership and career development to employee voice behaviour through engagement and commitment: evidence from state polytechnics in East Java, Indonesia. Cogent Bus. Manag. 12:2460628. doi: 10.1080/23311975.2025.2460628

[ref7001] Aguinis H. Beaty J. C. Boik R. J. Pierce C. A. (2005). Effect size and power in assessing moderating effects of categorical variables using multiple regression: a 30-year review. Journal of Applied Psychology 90:94. doi: 10.1037/0021-9010.90.1.94 15641892

[ref3] Al-Mahdy Y. F. H. Elwakil F. R. (2026). Empowering teachers for teaching innovation: roles of change leadership, AI self-efficacy, and innovative school climate. Sch. Leadersh. Manag. 46, 83–110. doi: 10.1080/13632434.2026.2618203

[ref4] Avalos B. (2011). Teacher professional development in teaching and teacher education over ten years. Teach. Teach. Educ. 27, 10–20. doi: 10.1016/j.tate.2010.08.007

[ref5] Bao Y. (2018). The influence mechanism of school leadership style on teachers' innovative work behavior: a mediation model based on teachers' innovative self-efficacy. Theor Pract. Educ. 38, 34–36.

[ref6] Blau P. M. (1964). Justice in social exchange. Sociol. Inq. 34, 193–206. doi: 10.1111/j.1475-682X.1964.tb00583.x

[ref7] Cao X. Peng C. Zhang W. (2021). Impact of transformational leadership on college teachers' change support behavior: a moderated mediation model. Stud. Psychol. Behav. 19, 273–279.

[ref8] Comstock M. Supovitz J. Kaul M. (2021). Exchange quality in teacher leadership ties: examining relational quality using social network and leader-member exchange theories. J. Prof. Cap. Community 6, 395–409. doi: 10.1108/jpcc-01-2021-0002

[ref9] Ding X. Xie X. (2026). The impact of vocational core competencies on entrepreneurial motivation among vocational college students: the mediating role of innovative self-efficacy and the moderating role of current educational level. Humanit. Soc. Sci. Commun. 13:128. doi: 10.1057/s41599-025-05983-6

[ref10] Du J. Chang H. (2018). Construction of teacher professional development model and factor analysis. Acad. Mon. Educ. 10, 48–56. doi: 10.16477/j.cnki.issn1674-2311.2018.10.005

[ref11] Durnali M. Gökbulut B. (2025). Empowering masters of creative problem solvers: the impact of STEM professional development training on teachers' attitudes, self-efficacy, and problem-solving skills. J. Intelligence 13:132. doi: 10.3390/jintelligence13100132, 41149771 PMC12565693

[ref12] Fornell C. Larcker D. F. (1981). Structural Equation Models with Unobservable Variables and Measurement Error: Algebra and Statistics. Journal of Marketing Research, vol. 18 American Marketing Association, 18, 382–388. doi: 10.1177/002224378101800313

[ref13] García-Martínez I. Pérez-Ferra M. Jiménez J. L. U. Quijano-López R. (2019). Promoting professional development for teachers through a scale of competence assessment. Res. Soc. Sci. Technol. 4, 147–162. doi: 10.46303/ressat.04.02.11

[ref14] Gong J. (2025). The effects of distributed leadership on teaching innovation in Shanghai, China: the mediating roles of teacher autonomy, teacher collaboration, and teacher self-efficacy. Front. Psychol. 16:1562838. doi: 10.3389/fpsyg.2025.1562838, 40727058 PMC12301307

[ref15] Hair J. F. Anderson R. E. Tatham R. L. Black W. C. (1998). Multivariate Data Analysis. 5th Edn Upper Saddle River, New Jersey: Prentice-Hall International.

[ref16] Hayes A. F. (2017). Introduction to Mediation, Moderation, and Conditional Process Analysis: A Regression-based Approach. Guilford Publications.

[ref17] Hebei Provincial Bureau of Statistics (2024). Hebei Statistical Yearbook. Shijiazhuang: China Statistics Press.

[ref18] Karacabey M. F. Bellibaş M. Ş. Adams D. (2022). Principal leadership and teacher professional learning in Turkish schools: examining the mediating effects of collective teacher efficacy and teacher trust. Educ. Stud. 48, 253–272. doi: 10.1080/03055698.2020.1749835

[ref19] Karakus M. Toprak M. Chen J. (2024). Demystifying the impact of educational leadership on teachers' subjective well-being: a bibliometric analysis and literature review. Educ. Manag. Adm. Leadersh. 54:17411432241242629. doi: 10.1177/17411432241242629

[ref20] Khan J. Jaafar M. Javed B. Mubarak N. Saudagar T. (2020). Does inclusive leadership affect project success? The mediating role of perceived psychological empowerment and psychological safety. Int. J. Manag. Proj. Bus. 13, 1077–1096. doi: 10.1108/ijmpb-10-2019-0267

[ref21] Kilag O. K. T. Sasan J. M. (2023). Unpacking the role of instructional leadership in teacher professional development. Adv. Qual. Res. 1, 63–73. doi: 10.31098/aqr.vli1.1380

[ref22] Kundu A. Roy D. D. (2023). How do teachers innovate? Role of efficacy for innovation and school climate perception. Psychol. Sch. 60, 4885–4903. doi: 10.1002/pits.22987

[ref23] Layek D. Koodamara N. K. (2024). Motivation, work experience, and teacher performance: a comparative study. Acta Psychol. 245:104217. doi: 10.1016/j.actpsy.2024.104217, 38493713

[ref24] Li X. Pei X. Zhao J. (2025). Intrinsic motivation and self-efficacy as pathways to innovative teaching: a mixed-methods study of faculty in Chinese higher education. BMC Psychol. 13:859. doi: 10.1186/s40359-025-03177-y, 40754519 PMC12320308

[ref25] Liang H. Lean H. H. Vasudevan A. (2026). How and when empowering leadership influences job performance: a dual-mediator model. PLoS One 21:e0332545. doi: 10.1371/journal.pone.0332545, 41686861 PMC12904590

[ref26] Liu Y. Fuller B. Hester K. Bennett R. J. Dickerson M. S. (2018). Linking authentic leadership to subordinate behaviors. Leadersh. Organ. Dev. J. 39, 218–233. doi: 10.1108/lodj-12-2016-0327

[ref27] Ma H. (2011). Mechanism and strategy of principal leadership promoting teacher professional development. J. Chin. Soc. Educ. 3, 41–43.

[ref28] Madison K. Eva N. De Cieri H. Goh Z. (2025). Social exchange theory in leadership research: a problematizing review. Leadersh. Q. 36:101924. doi: 10.1016/j.leaqua.2025.101924

[ref29] Maruyama G. M. (1998). Basics of Structural Equation Modeling. Thousand Oaks, CA: Sage.

[ref30] Modliba R. Fischer S. B. Treffers T. Welpe I. M. (2024). Translating leader–member exchange to innovative work behaviour: the role of creative self-efficacy and team support for innovation. Creat. Innov. Manag. 33, 671–684. doi: 10.1111/caim.12613

[ref31] Morgeson F. P. DeRue D. S. Karam E. P. (2010). Leadership in teams: a functional approach to understanding leadership structures and processes. J. Manag. 36, 5–39. doi: 10.1177/0149206309347376

[ref32] Mubarak N. Khan J. Yasmin R. Osmadi A. (2021). The impact of a proactive personality on innovative work behavior: the role of work engagement and transformational leadership. Leadersh. Organ. Dev. J. 42, 989–1003. doi: 10.1108/lodj-11-2020-0518

[ref33] Mubarak N. Salami B. Noor S. (2025). The curse to project innovation; role of passive leadership. IEEE Eng. Manag. Rev., 1–45. doi: 10.1109/emr.2025.3633929

[ref34] Prenger R. Poortman C. L. Handelzalts A. (2017). Factors influencing teachers' professional development in networked professional learning communities. Teach. Teach. Educ. 68, 77–90. doi: 10.1016/j.tate.2017.08.014

[ref35] Ren L. Shen H. (2024). The relationship between servant leadership and team innovation performance: mediating effect of self-efficacy. Heliyon 10:e27723. doi: 10.1016/j.heliyon.2024.e27723, 38509912 PMC10951603

[ref36] Safdar S. Faiz S. Mubarak N. (2024). Leadership dynamics in nursing: a comparative study of paternalistic approaches in China and Pakistan. Leadersh. Health Serv. 37, 570–586. doi: 10.1108/lhs-03-2024-0028, 39344570

[ref7002] Schumacker R. E. Lomax R. G. (2004). A beginner’s guide to structural equation modeling. Psychology Press.

[ref37] Schunk D. H. DiBenedetto M. K. (2020). Motivation and social cognitive theory. Contemp. Educ. Psychol. 60:101832. doi: 10.1016/j.cedpsych.2019.101832

[ref38] Tao F. (2025). An analysis of the collectivist value Core of the modern form of Chinese civilization. J. Shenyang Norm. Univ. 49, 53–59. doi: 10.19496/j.cnki.ssxb.2025.06.012

[ref39] Tierney P. Farmer S. M. (2002). Creative self-efficacy: its potential antecedents and relationship to creative performance. Acad. Manag. J. 45, 1137–1148. doi: 10.5465/3069429

[ref40] Vaiopoulou J. Gkontelos A. Stamovlasis D. (2026). Nonlinearity in innovative work behavior in educational systems: the dynamic interplay between creative self-efficacy and burnout. Teach. Teach. Educ. 175:105445. doi: 10.1016/j.tate.2026.105445

[ref41] Van Dijk E. E. Van Tartwijk J. Van Der Schaaf M. F. Kluijtmans M. (2020). What makes an expert university teacher? A systematic review and synthesis of frameworks for teacher expertise in higher education. Educ. Res. Rev. 31:100365. doi: 10.1016/j.edurev.2020.100365

[ref43] Wang H. Nie Q. (2021). Impact of inclusive leadership on employee innovative behavior: roles of employee trust and innovative self-efficacy. J. Chongqing Technol. Bus. Univ. 14, 1–12.

[ref44] Wu M. (2010). Operation and Application of AMOS. Chongqing: Chongqing University Press.

[ref45] Wu C. Ke Y. On the value of the educator Spirit for the professional development of international Chinese language teachers and its realization mechanism. J. Shandong Norm. Univ., (2026) 1–11. Available online at: https://link.cnki.net/urlid/37.1066.C.20260228.1235.002 (Accessed March 10, 2026).

[ref46] Xiong Y. Zhu X. (2013). Analysis of factors affecting teacher professional development. Theory Pract. Educ. 15, 42–43.

[ref47] Xu Z. Wang H. Suntrayuth S. (2022). Organizational climate, innovation orientation, and innovative work behavior: the mediating role of psychological safety and intrinsic motivation. Discret. Dyn. Nat. Soc. 2022:9067136. doi: 10.1155/2022/9067136

[ref48] Xuecheng W. Iqbal Q. Saina B. (2022). Factors affecting employee's retention: integration of situational leadership with social exchange theory. Front. Psychol. 13:872105. doi: 10.3389/fpsyg.2022.872105, 35899015 PMC9309793

[ref49] Yang J. Li Y. Duan X. Zhao W. Wang T. (2024). Relationship between digital leadership and special education teachers' innovative behavior: mediating role of teacher agency and moderating role of organizational innovation climate. Chin. J. Spec. Educ. 12, 62–70.

[ref50] Yang Y. Zhou J. (2025). Relationship between teacher professional identity and professional development in applied undergraduate universities: the moderating role of school organizational culture. Vocat. Tech. Educ. 46, 74–80.

[ref51] Yao J. You Y. Zhu J. (2020). Principal-teacher management communication and teachers' job performance: the mediating role of psychological empowerment and affective commitment. Asia Pac. Educ. Res. 29, 365–375. doi: 10.1007/s40299-019-00490-0

[ref52] Zhang H. Leng X. Cheng B. (2015). Effect of shared leadership on team members' innovation performance: mediating role of self-efficacy. Leadersh. Sci. 4Z, 43–45. doi: 10.19572/j.cnki.ldkx.2015.11.014

[ref53] Zhang G. Zhang X. Wang Y. (2024). Perceived insider status and employees' innovative behavior: the role of knowledge sharing and organizational innovation climate. Eur. J. Innov. Manag. 27, 589–607. doi: 10.1108/ejim-03-2022-0123

[ref7003] Zheng J. J. Jin S. H. Ma G. Y. (2009). The measurement of organizational innovation climate and its moderating effect in the relationship between employees’ innovation ability and innovation performance. Acta Psychologica Sinica 41:1203. Available online at: https://journal.psych.ac.cn/acps/EN/Y2009/V41/I12/1203

